# Automated spinopelvic measurements on radiographs with artificial intelligence: a multi-reader study

**DOI:** 10.1007/s11547-025-01957-5

**Published:** 2025-01-26

**Authors:** Boj Friedrich Hoppe, Johannes Rueckel, Jan Rudolph, Nicola Fink, Simon Weidert, Wolf Hohlbein, Adrian Cavalcanti-Kußmaul, Lena Trappmann, Basel Munawwar, Jens Ricke, Bastian Oliver Sabel

**Affiliations:** 1 Department of Radiology, University Hospital, LMU Munich, Marchioninistr. 15, 81377 Munich, Germany; 2 Institute of Neuroradiology, University Hospital, LMU Munich, Munich, Germany; 3 Department of Orthopaedics and Trauma Surgery, Musculoskeletal University Center Munich (MUM), University Hospital, LMU Munich, Munich, Germany

**Keywords:** Artificial Intelligence, Deep Learning, Spinopelvic Measurements, Radiographs

## Abstract

**Purpose:**

To develop an artificial intelligence (AI) algorithm for automated measurements of spinopelvic parameters on lateral radiographs and compare its performance to multiple experienced radiologists and surgeons.

**Methods:**

On lateral full-spine radiographs of 295 consecutive patients, a two-staged region-based convolutional neural network (R-CNN) was trained to detect anatomical landmarks and calculate thoracic kyphosis (TK), lumbar lordosis (LL), sacral slope (SS), and sagittal vertical axis (SVA). Performance was evaluated on 65 radiographs not used for training, which were measured independently by 6 readers (3 radiologists, 3 surgeons), and the median per measurement was set as the reference standard. Intraclass correlation coefficient (ICC), mean absolute error (MAE), and standard deviation (SD) were used for statistical analysis; while, ANOVA was used to search for significant differences between the AI and human readers.

**Results:**

Automatic measurements (AI) showed excellent correlation with the reference standard, with all ICCs within the range of the readers (TK: 0.92 [AI] vs. 0.85–0.96 [readers]; LL: 0.95 vs. 0.87–0.98; SS: 0.93 vs. 0.89–0.98; SVA: 1.00 vs. 0.99–1.00; all p < 0.001). Analysis of the MAE (± SD) revealed comparable results to the six readers (TK: 3.71° (± 4.24) [AI] v.s 1.86–5.88° (± 3.48–6.17) [readers]; LL: 4.53° ± 4.68 vs. 2.21–5.34° (± 2.60–7.38); SS: 4.56° (± 6.10) vs. 2.20–4.76° (± 3.15–7.37); SVA: 2.44 mm (± 3.93) vs. 1.22–2.79 mm (± 2.42–7.11)); while, ANOVA confirmed no significant difference between the errors of the AI and any human reader (all *p* > 0.05). Human reading time was on average 139 s per case (range: 86–231 s).

**Conclusion:**

Our AI algorithm provides spinopelvic measurements accurate within the variability of experienced readers, but with the potential to save time and increase reproducibility.

**Supplementary Information:**

The online version contains supplementary material available at 10.1007/s11547-025-01957-5.

## Introduction

Spinopelvic parameters describe the balance of the spinal column and the pelvis, which is essential for human upright gait [1]. Their imbalance due to deformities, degeneration, or trauma can lead to severe instabilities, ranging from low back pain to complete immobility, causing enormous strain for individuals, families, and whole economies [2, 3].

Correct and reliable measurements of spinopelvic parameters on radiographs are fundamental for diagnosis and prognosis, and to guide and monitor conservative or surgical therapies [4–6]. However, manual measurements are time-consuming, error-prone, and show high inter-reader variability [7, 8].

Recent advances in artificial intelligence (AI) have seen algorithms to improve and fasten image acquisition [9, 10], and with diagnostic accuracies at par with, or even superior to medical specialists at a variety of tasks [11–16]. This has also raised interest in automating spinopelvic measurements with growing success [17–23]. Nevertheless, as a recent review pointed out [24], previous studies are still lacking multi-reader assessment of AI to better account for clinical reality, which has already been established successfully in different use cases, such as fracture detection [11, 12], or interpretation of chest X-rays [13, 14].

For the following study, our goal was to i) develop and train an AI model for automatic measurements of clinically relevant spinopelvic parameters on lateral radiographs of the spine, ii) include various cases from clinical reality with fractures and instrumentation, iii) validate the AI model in an interdisciplinary multi-reader setting, including both radiologists and surgeons experienced with these measurements.

## Materials and methods

### Study design

This study was approved by the institutional review board (approval number 18–399). Informed consent was waived due to the retrospective and non-interventional nature of the study. All procedures were conducted in accordance with the Declaration of Helsinki (as revised in 2013).

### Imaging data

All imaging data were queried retrospectively from our institutional Picture and Archiving System (PACS) for patients receiving lateral radiographs of the whole spine between September 2012 and May 2019. At our institution, these are acquired with the EOS System (EOS Imaging, Paris, France), where a vertically moving X-ray source allows for distortion-free images in a standing position under weight-bearing conditions [25], without the need for stitching conventional images and with very low radiation dose [26].

Inclusion criteria were: i) lateral images of the full spine in a standing position, and ii) acquisition ranging cranial from the external auditory canal and caudal to the femoral heads. Exclusion criteria were: ii) incomplete capturing of the spine, ii) motion artifacts during image acquisition, and iii) severe spinal deformities, defined by the presence of hemivertebrae or moderate to severe scoliosis (Cobb angle ≥ 25°), [27] which would cause superimposition artifacts on lateral images. Otherwise, cases with foreign material, e.g., from spinal instrumentation or hip replacement, and vertebral fractures were all included to represent clinical reality.

This resulted in a set of 295 images for algorithm development, randomly split into 80% training data and 20% for internal validation and optimization. Another randomly selected test set of 65 images was completely withheld from training and used for subsequent performance evaluation of the algorithm.

### Definition of spinopelvic measurements

Five frequently used clinically relevant spinopelvic measurements on lateral radiographs were used for the current study, as defined in the following. The thoracic kyphosis (TK) of the thoracic spine is theoretically considered the angle between T1 and T12, which is in practice often impossible to delineate due to the superposition of the humeral heads [1], and therefore in clinical routine and most previous studies alternatively measured from the superior endplate of T4 to the inferior endplate of T12 (normal: 30–50°) [28], which we adopted for our study. More agreement exists on the lumbar lordosis (LL), measured from the superior endplate of L1 to the superior endplate of S1 and denoted as negative values (normal: –60 to –50°) [28], the sacral slope (SS), measured between the superior endplate of S1 and a horizontal line (normal: 35–45°) [28], and the sagittal vertical axis (SVA), measured as the horizontal distance from the plumb line of the center of C7 to the superior endpoint of S1, with positive values in front of S1, and negative values behind (normal: ± 20 mm) [28].

### Algorithm development

Collected training data were annotated for anatomical landmarks by two extensively trained annotators on a dedicated internal platform. Annotations included the four corners of each vertebra (C2-S1, 94 labels per case, total of ~ 27.730 labels), and all labels were reviewed by a radiologist with strong experience in orthopedic radiographs. The annotated images were augmented manifold (including rotation, translation, cropping, resizing, blurring, and distortion) and used to train a two-staged neural network. First, a region-based convolutional neural network (R-CNN) was used to automatically detect the whole spine centers from the images in a single shot. Then, the original image was sliced into small areas containing the vertebrae based on the detected spinal centers, and a second R-CNN was used to automatically detect the four vertebral corners. Finally, the predicted landmarks were post-processed with geometric formulae to automatically calculate the above-mentioned spinopelvic angles and measurements and displayed over the input images to allow visual inspection of the results.

### Reference standard definition

Six readers independently measured the validation data set manually, after receiving a verbal and written introduction with all the specifications for the measurements. Readers included three radiologists (B.F.H, B.O.S., and J.Rue., with 3, 6, and 3 years of experience; referred to as “Readers R1-R3”) and three orthopedic surgeons (A.C.-K., S.W., and W.H., with 3, 8 and 5 years of experience; referred to as “Readers S1-S3”). As we found that even the most experienced reader would occasionally over- or underestimate a single reading (Supplemental Fig. S2), the median of the readers was used as the consensual reference standard. For an even number of values (from six readers), the median is defined as the arithmetic mean of the two middle values, when ordered from lowest to highest. This further implies that no single reader alone could establish the reference standard for any given case.

### Statistical analysis

Quantitative measurements were expressed as mean and standard deviation (± SD); while, categorical variables were expressed as counts and percentages. Normal distribution of the measurements was confirmed with histogram plots (Supplemental Fig. S1). Performance of the AI algorithm and the readers was evaluated using the mean absolute error (MAE) with standard deviation (SD), Pearson’s correlation (r) with the reference standard, Bland–Altman plots for mean difference visualization, and cumulative distribution function (CDF) to evaluate performance up to clinically relevant thresholds and calculate a normalized area under the curve (AUC) for further quantification. Intraclass correlation coefficient (ICC) was calculated based on a two-way random-effects model with absolute agreement [29]. Agreement was defined as previously described: ICC ≥ 0.90 as excellent, 0.90–0.75 as good, 0.75–0.50 as moderate, and ≤ 0.50 as poor [30].

**Fig. 1:**
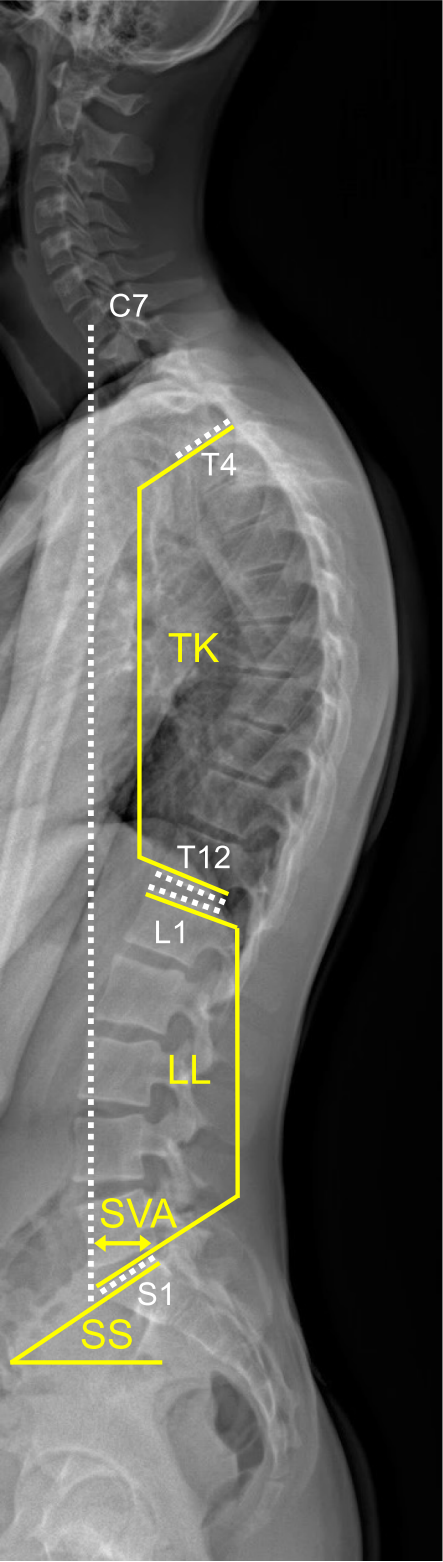
Examples of spinopelvic parameters and required landmarks. (TK): Thoracic kyphosis, angle measured from the superior endplate of T4 to the inferior endplate of T12 (normal: 30–50°). (LL): Lumbar lordosis, angle measured from the superior endplate of L1 to the superior endplate of S1 (normal: –50 to 60°). (SS): Sacral slope, angle measured between the superior endplate of S1 and a horizontal line (normal: 35–45°). (SVA): Sagittal balance, measured as the horizontal distance from the plumb line of the center of C7 to the superior endpoint of S1, with positive values in front of S1, and negative values behind (normal: ± 20 mm)

To test for any significant differences between AI and human readers, analysis of variance (ANOVA) of the errors was performed with Tukey’s post hoc test, if needed. All analysis and visualizations were performed in Python (Version 3.12, Python Software Foundation), with recent SciPy, statsmodels, and seaborn libraries. Two-sided significance testing was conducted with an α of 5% (*p* < 0.05) (Fig. 1).

## Results

### Study population

The evaluation cohort consisted of 65 patients (39 female; 60.0%), from all different age groups (mean age: 47.8 [± 24.1] years, range: 7–85 years), with a wide range of (pathologic) spinopelvic measurements. Over a third of patients had vertebral fractures (23; 35.4%), and about a quarter of patients had previously undergone spinal instrumentation (16; 24.6%), reflecting the broad spectrum of clinical reality (Table 1).

**Table 1:** Characteristics of the cohort used for evaluation

Variable (unit)	N or mean	% or ± SD	Range
*Patients*	65	(100.0%)	–
Age (years)	47.8	(± 24.1)	(7; 85)
Sex (female)	39	(60.0%)	–
Vertebral fractures (cases)	23	(35.4%)	–
Spinal instrumentation (cases)	16	(24.6%)	–
*Measurements*			
Thoracic kyphosis (°)	43.4	(± 14.8)	(14.6; 87.5)
Lumbar lordosis (°)	–51.9	(± 14.9)	(–89.1; –5.7)
Sacral slope (°)	36.0	(± 10.5)	(5.3; 55.3)
Sagittal vertical axis (mm)	30.8	(± 45.8)	(–63.4; 145.2)

– SD; standard deviation

### Algorithm performance

**Fig. 2:**
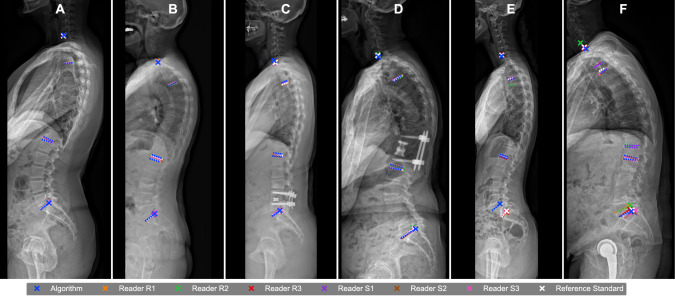
Examples of AI and human spinopelvic measurements. **A**-**D** Perfect agreement between all human readers and AI. The algorithm performed well even in cases with **B** vertebral fractures (wedge fracture L3), or with **C**, **D** dorsal instrumentation. **E**, **F** Landmarks were detected incorrectly, especially in cases with anatomical variation **E** or severe overlay, which also resulted in disagreement between the multiple readers (**F**)

**Fig. 3:**
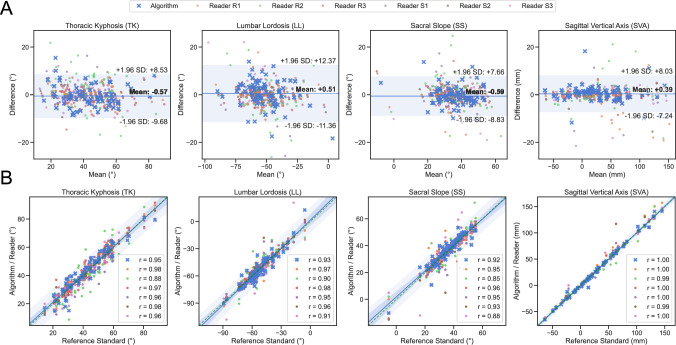
Performance analysis of AI and readers. Measurements, in columns from left to right: thoracic kyphosis (TK), lumbar lordosis (LL), sacral slope (SS), and sagittal vertical axis (SVA). **A** Bland–Altman plots, showing a low mean difference for AI with no proportional bias. Horizontal line *(blue)* marks mean, horizontal corridor *(light blue)* marks ± 1.96 standard deviations (SD). **B** Regression analysis of AI and readers vs. reference standard, showing excellent correlation of measurements with the reference standard. Diagonal dashed line *(green)* shows theoretical perfect fit, diagonal straight line *(blue)* shows the fit for AI measurements, diagonal corridor *(light blue)* shows 95% confidence interval of prediction limits for AI; labels show Pearson’s correlation coefficient (“r”) for AI and each reader

All 65 cases were successfully processed by the AI (100.0% success rate), including special cases with vertebral fractures, deformities, or spinal instrumentation (examples in Fig. 2). AI analysis took a maximum of 1 s per case; while, human readers took an average of 139 s per case (range: 86–231 s). Bland–Altman plots showed a low mean difference for all automatic measurements (TK: −0.57; LL: 0.51; SS: 0.59; SVA: 0.39), with no proportional bias (Fig. 3A).

All AI-based results had an excellent correlation with the reference standard (ICC: 0.92–1.00; all *p* < 0.001), with the highest result for sagittal vertical axis (SVA), and the lowest result for thoracic kyphosis (TK), but for every measurement, the ICC was within the range of the six human readers (Table 2, Fig. 3B). Further, the mean absolute error (MAE) was also within the range of the readers for all four measurements, with the smallest errors for sagittal vertical axis (AI: 2.44 mm [± 3.93]; readers: 1.22–2.79 mm), followed by thoracic kyphosis (AI: 3.14° [± 4.24]; readers: 1.97–3.87°), lumbar lordosis (AI: 3.71° [± 4.68]; readers: 1.86–5.88°), and sacral slope (AI: 4.56° [± 6.10]; readers: 2.20–4.76°) (Table 2).

**Table 2:** Performance evaluation of the AI and the readers

	Thoracic kyphosis (TK)			Lumbar lordosis (LL)			Sacral slope (SS)			Sagittal vertical axis (SVA)		
	MAE	SD	ICC	MAE	SD	ICC	MAE	SD	ICC	MAE	SD	ICC
Algorithm	3.14	[± 4.24]	0.92	3.71	[± 4.68]	0.95	4.56	[± 6.10]	0.93	2.44	[± 3.93]	1.00
Reader R1	2.07	[± 3.48]	0.94	2.19	[± 2.83]	0.98	2.21	[± 3.60]	0.97	2.41	[± 4.74]	0.99
Reader R2	3.87	[± 6.17]	0.85	5.88	[± 7.38]	0.87	4.76	[± 7.03]	0.90	2.79	[± 5.24]	0.99
Reader R3	1.98	[± 3.07]	0.96	3.17	[± 3.73]	0.96	2.25	[± 3.15]	0.98	1.22	[± 2.42]	1.00
Reader S1	2.26	[± 3.74]	0.94	2.94	[± 4.15]	0.96	2.86	[± 4.81]	0.95	1.70	[± 3.49]	1.00
Reader S2	1.97	[± 4.36]	0.92	1.86	[± 2.60]	0.98	2.20	[± 4.52]	0.96	2.23	[± 7.11]	0.99
Reader S3	3.31	[± 5.79]	0.87	3.31	[± 4.25]	0.96	4.49	[± 7.37]	0.89	2.09	[± 4.05]	1.00

– MAE; mean absolute error, SD; standard deviation, ICC; intraclass correlation coefficient

All these errors were below the threshold of < 5°, which has been shown previously as the normal inter-reader variability and is generally regarded as acceptable [8, 31].

**Fig. 4:**
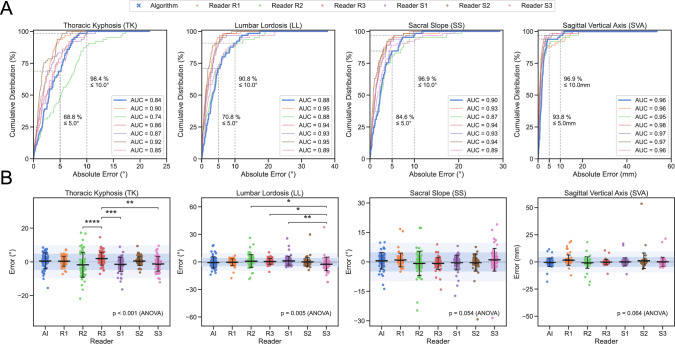
Error analysis of AI and readers. Measurements, in columns from left to right: thoracic kyphosis (TK), lumbar lordosis (LL), sacral slope (SS) and sagittal vertical axis (SVA). **A** Cumulative distribution function (CDF), plotting incremental percentage of cases *(y-axis)* below a certain absolute error *(x-axis)*. Intersections *(dashed gray lines)* show percentage of cases for AI with errors below clinically relevant thresholds (5 mm/10 mm; 5°/10°). Labels show area under the curve (AUC) of the normalized maximum for quantitative comparison. **B** Stripplots of error for AI and readers, showing no significant difference between AI and any human reader, only among some human readers for TK and LL. Horizontal bars show mean ± SD. Horizontal corridors *(blue)* mark error of ± 5°/mm and ± 10°/mm *(light blue)*. (* p < 0.05; ** p < 0.01; *** p < *0.001)*

Cumulative distribution function (CDF) enabled more detailed analysis, by incrementally plotting the percentage of cases (y-axis) below a certain absolute error (x-axis), thereby allowing clinically relevant thresholds to be read and calculating a normalized area under the curve (AUC) for quantitative comparison (Fig. 4A). This revealed on a per case analysis, that an error < 5° was achieved in 84.6% of cases for SS, in 70.8% for LL, and in 68.8% for TK; while, a higher threshold of < 10° (as also utilized in previous studies [18]) was reached in well over 90% of cases for all measurements (SS: 96.9%; LL: 90.8%; TK: 98.4%).

Analysis of variance (ANOVA) showed no significant difference between the errors of the AI and the human readers for SS (p = 0.054) and SVA (p = 0.064), but for TK (*p* < 0.001) and LL (*p* < 0.005). However, post hoc Tukey’s test revealed no significant differences between AI and any of the human readers, only for some readers between each other (TK: R3 vs. R2, S2, and S3; LL: S3 vs. S1, R2, and R3; all p < 0.05; full results in Supplemental Table S1) (Fig. 4B).

## Discussion

In this study, we developed an AI algorithm to measure clinically relevant spinopelvic parameters on radiographs and validated it in an interdisciplinary multi-reader setting with six experienced physicians, equally consisting of radiologists and surgeons.

All automatic measurements (TK, LL, SS, and SVA) showed excellent correlations with the reference standard, and deviations were all within the range of the readers. Further, no significant differences were found between the errors of the AI to those of the multiple human readers, thereby placing the AI within the field of the naturally occurring differences of multiple clinicians.

Specifically, the mean absolute error was < 5° for all AI-based angles, which has been shown previously as the normal inter-reader variability and is generally regarded acceptable [8, 31]. In-depth analysis showed, that this threshold was undercut in 70–80% of cases, underlining clinical usability. Further, we used a comprehensive validation pipeline including various statistical tests, which offers a high transparency of the results.

Our model was able to analyze all test cases (success rate 100%), including those with deformities, fractures, or spinal instrumentation, due to the diverse data used for training, increasing clinical value even for challenging cases. In contrast, a previous study reported a success rate of 84% [20], and even in a recent publication [22], some cases failed automatic analysis. Further, even most recent studies only focused on normal cases and excluded spinal pathologies or foreign materials [20–22]; while, their strict case selection may have led to better performance in a laboratory setting, their inability to process pathologic cases minimizes clinical value.

Unsurprisingly, automatic measurements only took a fraction of the reading time (< 1 s); while, humans spend on average over 2 min per case. Given the possibility for a human reader to visually counter check a render of the AI-detected landmarks within seconds, this is an excellent use case of “explainable AI” [32] and holds great potential to shorten reading times. Also, automatic analysis over time could identify any abnormal changes and thus help to flag cases suspicious for, e.g., new fractures or failing therapies [5, 6]. For example, a recent meta-analysis proved a significant increase for TK and SVA with the occurrence of osteoporotic vertebral fractures [33]; while, LL and SVA have been shown to be predictive of distal junctional failure after corrective surgery of osteoporotic vertebral fractures [34], and a higher increase in lordosis after lumbar fusion was related to post-operative L5 radiculopathy [35].

In today’s world, where the gap between the steadily increasing number of medical procedures and the stagnating number of medical professionals is widening, resulting in alarming stress levels and burn-out rates [36], AI-based support systems are becoming more a necessity than an option. As multiple recent studies showed, physicians can clearly benefit from AI-systems [11–14, 37, 38], and through continuous use they can build trust toward an AI, by understanding its’ capabilities and weaknesses, and use the systems effectively [39].

Also, we saw reduced AI performance in a few cases, e.g., with lumbar variations or severe deformities and resulting artifacts—however, these cases also remained challenging for human readers and are known to result in a higher inter-reader-variability in clinical practice. Yet our approach to set the median of the readers as the reference standard (which for an even number of values, such as our six readers, is defined as the mean of the two middle values), also showed a robust reference standard in demanding cases, as every single reader had a relevant number of outliers, which would have led to a false reference standard, but was mitigated by our method.

As one of our study’s biggest strengths, we see our extended interdisciplinary multi-reader comparison, which allowed us to prove the algorithm comparable to multiple clinically experienced experts.

So far, nearly all previous studies only chose a single radiologist or surgeon for the reference standard and comparison [17, 18, 40]. In notable exceptions, a single resident was reviewed by a single senior radiologist [22], or a second reader measured at least half of the cases [23], and only one group so far included three surgeons in comparison [19, 20]. To the best of our knowledge, no study yet has made the effort to include more than three readers; while, we analyzed a total of six readers, consisting equally of experienced radiologists and surgeons.

Our study had limitations, including the retrospective and single-center character. Nevertheless, the proposed model could be used as a starting point for further investigation, e.g., utilizing a federated learning approach with prospective or multi-center data, for re-training and further optimization. Further, we only included data from the EOS System, which has been shown superior to conventional radiographs for higher image quality and lower radiation exposure [25, 26], but may limit the generalizability of our model—however, the deep learning techniques used (CNN) enable transfer learning and the applicability to unseen conventional radiographs should be investigated in future studies. Another limitation could be seen in our relatively small dataset of 295 training cases. However, modern pre-trained models and effective augmentation techniques enable generalizability even with little training data, especially if this represents a broad spectrum of variation (as in our case), and notable performance was previously achieved with comparable [41], or even smaller datasets [18].

In conclusion, we showed that our AI algorithm provides spinopelvic measurements accurate within the variability of multiple experienced readers, but with the potential to save time and increase reproducibility. Future studies should extend these works and further evaluate the clinical impact of AI-assisted reading.

## Supplementary information

Below is the link to the electronic supplementary material.

Supplementary file1 (DOCX 283 KB)

## Data Availability

The datasets used and/or analyzed during the current study are available from the corresponding author upon reasonable request. **Disclosures**: The mentioned artificial intelligence prototype algorithm is not yet commercially available, future availability cannot be guaranteed.

## Abbreviations

AI: Artificial intelligence
ANOVA: Analysis of variance
AUC: Area under the curve
ICC: Intraclass correlation coefficient
LL: Lumbar lordosis
MAE: Mean absolute error
SVA: Sagittal vertical axis
SD: Standard deviation
SS: Sacral slope
TK: Thoracic kyphosis
