# Supplementary material for: Automated spinopelvic measurements on radiographs with artificial intelligence: a multi-reader study
Source: Radiol Med. 2025 Jan 26;130(3):359–67. doi: 10.1007/s11547-025-01957-5 (PMC11903605; doi:10.1007/s11547-025-01957-5)
Supplement: Supplementary file 1 — Supplementary file1 (DOCX 283 KB) [file 11547_2025_1957_MOESM1_ESM.docx]

# Supplemental Materials:

**Supplemental Figure S1: Histogram of errors shows normal distribution for readers and AI.** Values (binned) on the x-axis, counts on the y-axis.

**Supplemental Figure S2: Heatmap of case-based errors per reader.** Error per case for each measurement by every reader is color-coded, brighter colors indicate larger errors (labeled colormap on the right side of each plot). These show, that every reader may occasionally measure erroneous outliers, and a “swarm intelligence” outperforms a “single expert only” approach, hence the median of the readers was chosen as the ground truth, which for an even number of readers is defined as the arithmetic mean of the two middle values.

**Supplemental Table S1: Results from Post-hoc Tukey test of ANOVA of errors.** Only results comparing AI to human readers, or any significant results are shown.

| **Kyphosis Angle**  ANOVA: p < 0.001 (***) | | | |
| --- | --- | --- | --- |
| **Reader A** | **Reader B** | **p** |  |
| **AI** | R1 | 1.000 | ns |
| **AI** | R2 | 0.062 | ns |
| **AI** | R3 | 0.580 | ns |
| **AI** | S1 | 0.141 | ns |
| **AI** | S2 | 1.000 | ns |
| **AI** | S3 | 0.267 | ns |
|  |  |  |  |
| R2 | R3 | < 0.001 | *** |
| R3 | S1 | < 0.001 | *** |
| R3 | S3 | 0.001 | ** |

| **Lordosis Angle**  ANOVA: p < 0.005 (**) | | | |
| --- | --- | --- | --- |
| **Reader A** | **Reader B** | **p** |  |
| **AI** | R1 | 1.000 | ns |
| **AI** | R2 | 0.671 | ns |
| **AI** | R3 | 0.875 | ns |
| **AI** | S1 | 0.467 | ns |
| **AI** | S2 | 0.992 | ns |
| **AI** | S3 | 0.539 | ns |
|  |  |  |  |
| R2 | S3 | 0.012 | * |
| R3 | S3 | 0.037 | * |
| S1 | S3 | 0.004 | ** |
